# Supplementary material for: Improvements in life expectancy mask rising trends in heat-related excess mortality attributable to climate change
Source: Nat Commun. 2025 Nov 26;16:11632. doi: 10.1038/s41467-025-66681-0 (PMC12748886; doi:10.1038/s41467-025-66681-0)
Supplement: Supplementary file 1 — Supplementary Information [file 41467_2025_66681_MOESM1_ESM.pdf]

Supplementary Information for

Improvements in life expectancy mask rising trends in heat-related excess mortality attributable to climate change

Veronika Huber[1,2,3]*, Susanne Breitner-Busch[2,3], Hanna Feldbusch[3], Katja Frieler[4,5], Cheng He[3], Franziska Matthies-Wiesler[3], Matthias Mengel[4], Siqi Zhang[6], Annette Peters[2,3,7], Alexandra Schneider[3]

[1] Doñana Biological Station (EBD), Spanish National Research Council (CSIC), Sevilla, Spain

[2] Chair of Epidemiology, Institute for Medical Information Processing, Biometry, and Epidemiology, Faculty of Medicine, Ludwigs-Maximilian-Universität (LMU), Munich, Germany

[3] Institute of Epidemiology, Helmholtz Zentrum München – German Research Center for Environmental Health, Neuherberg, Germany

[4] Potsdam Institute for Climate Impact Research, Potsdam, Germany

[5] Institute of Environmental Science and Geography, University of Potsdam, Potsdam, Germany

[6] Department of Environmental Health Sciences, Yale School of Public Health, New Haven, CT, USA

[7] Munich Heart Alliance, German Center for Cardiovascular Health (DZHK e.V., partner-site Munich), Munich, Germany

*corresponding author: veronika.huber@ebd.csic.es

Table of content

Supplementary Table 1 3
Supplementary Table 2 4
Supplementary Table 3 5
Supplementary Table 4 6
Supplementary Table 5 7
Supplementary Table 6 8
Supplementary Table 7 9
Supplementary Fig. 1 10
Supplementary Fig. 2 11
Supplementary Fig. 3 12
Supplementary Fig. 4 13
Supplementary Fig. 5 14
Supplementary Fig. 6 15
Supplementary Fig. 7 16
Supplementary Fig. 8 17
Supplementary Fig. 9 18
Supplementary Fig. 10 19
Supplementary Fig. 11 20
Supplementary Fig. 12 23
Supplementary Fig. 13 21
Supplementary Fig. 14 22

Supplementary Table 1 Summary statistics for 15 major German cities from 1993 to 2022.

City name	Cumulative death count in the warm season (Jun-Sep)	Mean warm-season temperature (°C)	Mean population	Mean share in total German population (%)
Berlin	336,022	18.0	3,418,000	4.2
Bremen	63,808	16.9	550,000	0.7
Cologne	95,533	17.5	1,009,000	1.2
Dortmund	64,511	17.1	585,000	0.7
Dresden	53,466	17.3	511,000	0.6
Duisburg	57,297	17.5	505,000	0.6
Dusseldorf	65,396	17.5	588,000	0.7
Essen	78,730	17.1	586,000	0.7
Frankfurt	69,075	18.1	680,000	0.8
Hamburg	184,299	16.9	1,735,000	2.1
Hannover	116,404	17.2	1,119,000	1.4
Leipzig	65,306	17.9	525,000	0.6
Munich	121,202	16.8	1,32,9000	1.6
Nuremberg	58,047	17.5	494,000	0.6
Stuttgart	56,883	17.2	595,000	0.7
All cities	1,485,979	17.4	14,229,000	17.3

Supplementary Table 2 Information on demographic and socio-economic indicators sourced from the INKAR database.

Variable	Data coverage	Extrapolated years
population	1995-2020	1993-1994, 2021-2022
woman.quota (%)	1995-2020	1993-1994, 2021-2022
residents.over.65 (%)	1995-2020	1993-1994, 2021-2022
life.expectancy at birth (years)	1995-2020	1993-1994
foreign.residents (%)	1995-2020	1993-1994, 2021-2022
household.income (€)	2000-2019	1993-1999, 2020-2022
population.density (km^{-2})	1996, 2000, 2004, 2008-2020	None
unemployment (%)	1995-2020	1993-1994, 2021-2022

Supplementary Table 3 Second-stage longitudinal mixed-effect meta-regression models. We report p-values of likelihood ratio tests (LRT) for single term deletion of included meta-predictors. The model marked with an asterisk was used to pool temperature-mortality associations and to compute best-linear unbiased predictors (BLUPs) for individual cities.

Model		AIC	BIC	I^2 (%)	Cochran Q-test p-value	Single term deletion p-value of LRT	
						ns(year)	other meta-predictors
Model 0	(1|city)	114	135	38.1	<0.001		
Model 0.1	(1|eastwest/city)	120	152	38.1	<0.001		
Model 0.2	(1|fstate/city)	112	145	38.1	<0.001		
Model 0.3	+ ns(year) (1|city)	89	132	34.5	<0.001	<0.001	
Model 0.4	+ ns(year) (1+ns(year)|city)	95	160	34.5	<0.001	0.0074	
	Model 0.3						
Model 1	+ average.annual.temp	86	139	27.5	<0.001	0.0037	0.028
Model 2	+ range.temp	92	146	35.1	<0.001	<0.001	0.39
Model 3	+ average.summer.temp	86	140	32.3	<0.001	0.031	0.039
Model 4	+ heat.alert.days	81	135	33.9	<0.001	0.0012	0.004
Model 5	+ population	93	147	34.8	<0.001	<0.001	0.66
Model 6	+ women.quota	91	145	34.6	<0.001	0.035	0.36
Model 7	+ residents.over.65	91	145	34.8	<0.001	<0.001	0.35
Model 8	+ life.expectancy	82	136	29.6	<0.001	0.068	0.0049
Model 9	+ foreign.residents	92	146	34.7	<0.001	<0.001	0.54
Model 10	+ household.income	93	147	32.5	<0.001	0.13	0.58
Model 11	+ population.density	94	149	33.1	<0.001	<0.001	0.99
Model 12	+ perc.unemployed	86	140	29.6	<0.001	0.0044	0.032
Model 13	+ average.population.age	88	142	34.3	<0.001	<0.001	0.081
Full model	+ all meta-predictors	105	288	23.5	<0.001		
Backward selection	Equivalent to model 0.3						
Step forward selection*	+ average.annual.temp<br>+ heat.alert.days<br>+ life.expectancy<br>+ average.population.age	77	163	24.6	<0.001	0.013	0.070<br>0.043<br>0.064<br>0.094

Supplementary Table 4 Linear slope estimates (with 95% confidence intervals) of city-average temporal trends in (climate-change attributable) heat-related excess deaths from 1993 to 2022. Same as Table 2, but for attributable numbers (AN) instead of attributable fractions (AF).

Temporal trend in heat-related excess mortality		
	With LE improvements	W/o LE improvements
AN (with CC) (deaths year^{-1})	-15 (-57, 28)	44 (-10, 98)
AN (w/o CC) (deaths year^{-1})	-19 (-46, 8)	2 (-31, 35)
Temporal trend in heat-related excess mortality attributable to climate change		
	With LE improvements	W/o LE improvements
AN_{CC} (deaths year^{-1})	5 (-12, 21)	42 (19, 66)
P_{CC} (% decade^{-1})	5.6 (2.6, 8.6)	5.9 (2.7, 9.1)

CC: climate change; AN: attributable number; AN_{CC}: attributable number due to climate change; P_{CC}: relative proportion due to climate change

Supplementary Table 5 Sensitivity analyses: Linear slope estimates (with 95% CI) of city-average temporal trends (change in % points per decade) from 1993 to 2022 for different modelling approaches.

Modelling approach	With LE improvements				W/o LE improvements			
	AF (with CC)	AF (w/o CC)	AF_{CC}	P_{CC}	AF (with CC)	AF (w/o CC)	AF_{CC}	P_{CC}
Default	-0.3 (-1.1, 0.5)	-0.4 (-0.9, 0.1)	0.1 (-0.3, 0.4)	5.6 (2.6, 8.6)	0.8 (-0.2, 1.8)	0.0 (-0.6, 0.7)	0.8 (0.3, 1.2)	5.9 (2.6, 9.1)
Moving average GMST	-0.3 (-1.1, 0.5)	-0.4 (-1.0, 0.1)	0.1 (-0.2, 0.4)	6.4 (3.3, 9.5)	0.8 (-0.2, 1.8)	0.0 (-0.7, 0.6)	0.8 (0.4, 1.2)	6.6 (3.2, 10.0)
Averaged daily mortality	-0.3 (-1.0, 0.4)	-0.4 (-0.8, 0.1)	0.1 (-0.2, 0.4)	5.7 (2.8, 8.5)	0.8 (-0.2, 1.7)	0.0 (-0.5, 0.6)	0.7 (0.3, 1.2)	6.0 (2.9, 9.1)
Five 6-year subperiods	-0.1 (-0.9, 0.7)	-0.3 (-0.8, 0.2)	0.2 (-0.1, 0.5)	6.2 (3.1, 9.3)	0.8 (-0.2, 1.8)	0.0 (-0.6, 0.6)	0.8 (0.4, 1.3)	6.9 (3.2, 10.7)
Without 2003	-0.1 (-0.9, 0.6)	-0.3 (-0.7, 0.2)	0.1 (-0.2, 0.4)	5.4 (2.7, 8.1)	1.1 (0.1, 2.1)	0.1 (-0.5, 0.7)	0.9 (0.5, 1.4)	6.1 (2.9, 9.3)

Supplementary Table 6 Maximum climate-change attributable annual heat-related death burden (with 95% eCI) from 1993 to 2022, accounting for observed changes in population susceptibility to heat (i.e., with LE improvements). The year when the respective maximum in annual estimates was observed is also given.

City name	Maximum attributable annual heat excess deaths (AN_{CC}) (95% eCI)	Year	Maximum attributable heat excess mortality fraction (AF_{CC}, %) (95% eCI)	Year	Maximum relative attributable proportion (P_{CC}, %) (95% eCI)	Year
Berlin	477 (418, 534)	2006	4.5 (3.95, 5.04)	2006	77.7 (71.6, 94.7)	2009
Bremen	58 (44, 72)	2003	2.72 (2.02, 3.36)	2006	81.2 (78.1, 89.2)	2017
Cologne	152 (128, 172)	2003	4.66 (3.95, 5.30)	2003	67.9 (64.8, 74.9)	2021
Dortmund	85 (69, 99)	2003	3.95 (3.20, 4.62)	2003	73.7 (70.8, 79.8)	2017
Dresden	53 (23, 82)	2018	2.82 (2.05, 3.57)	2006	76.8 (68.6, 93.2)	2020
Dusseldorf	85 (63, 104)	2018	4.29 (3.53, 4.97)	2006	70.1 (67.2, 75.5)	2009
Duisburg	102 (84, 118)	2003	4.27 (3.52, 4.98)	2003	70.5 (67.6, 76.1)	2017
Essen	92 (67, 116)	2018	3.38 (2.69, 4.04)	2006	73.1 (71.1, 76.9)	2017
Frankfurt	105 (82, 126)	2003	4.25 (3.33, 5.10)	2003	76.2 (62.6, 81.4)	2022
Hamburg	147 (113, 178)	2006	2.52 (1.94, 3.05)	2006	87.3 (81.4, 98.9)	2017
Hannover	110 (85, 132)	2003	2.77 (2.15, 3.32)	2003	83.9 (79.6, 95.9)	2017
Leipzig	82 (52, 114)	2018	3.57 (2.61, 4.47)	2003	71.8 (62.9, 86.0)	2020
Munich	136 (88, 183)	2003	3.24 (2.10, 4.35)	2003	80.9 (72.7, 93.9)	2020
Nuremberg	74 (57, 91)	2003	3.69 (2.83, 4.51)	2003	71.9 (69.7, 76.1)	2021
Stuttgart	87 (64, 109)	2015	4.24 (3.10, 5.30)	2015	74.2 (70.3, 88.0)	2021
All cities	1746 (1403, 2072)	2003	3.52 (2.83, 4.17)	2003	69.4 (62.5, 77.1)	2022

eCI: empirical confidence interval

Supplementary Table 7 Sensitivity analyses: Climate-change attributable heat-related death burden pooled across cities over 1993-2022 for different modelling approaches, accounting for observed changes in population susceptibility to heat, i.e. improvements in LE (cf. Table 1).

Modelling approach	Sum of attributable heat excess deaths in 1993-2022 (95% eCI)	Attributable annual heat excess deaths (AN_{CC}) (95% eCI)	Attributable heat excess mortality fraction (AF_{CC}, %) (95% eCI)	Relative attributable proportion (P_{CC}, %) (95% eCI)
Default	28506 (20758, 36363)	950 (692, 1212)	1.92 (1.40, 2.45)	53.6 (49.8, 58.9)
Moving average GMST	27698 (20091, 35481)	923 (670, 1183)	1.86 (1.35, 2.39)	52.1 (48.7, 56.8)
Averaged daily mortality	27681 (20029, 35550)	923 (668, 1185)	1.86 (1.35, 2.39)	54.3 (50.3, 59.8)
Five 6-year subperiods	25658 (18778, 33033)	855 (626, 1101)	1.73 (1.26, 2.22)	53.3 (50.3, 57.4)
Without 2003	-*	889 (646, 1125)	1.79 (1.3, 2.27)	54.7 (50.7, 60.2)

* Not comparable because one year missing

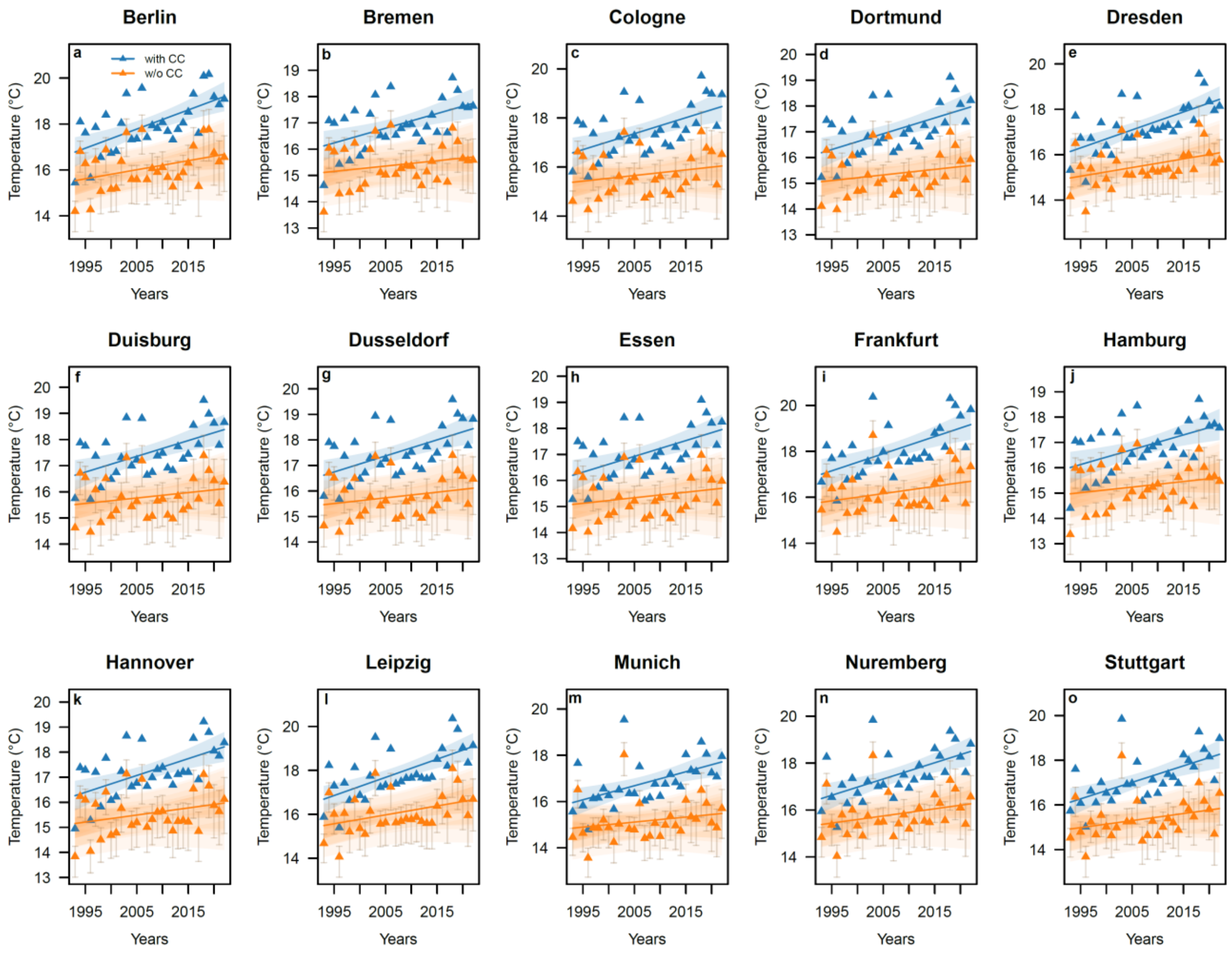

Supplementary Fig. 1 Factual (blue) and counterfactual (orange) average warm-season temperatures by city with linear regression lines (shading: 2*standard errors); error bars (light shading) correspond to the uncertainty range (min, max) in counterfactual temperatures (trends), based on best estimates, lower and upper bounds of 95% confidence intervals for estimates of GMST and local scaling coefficients (n=9 counterfactual temperature series); CC: climate change

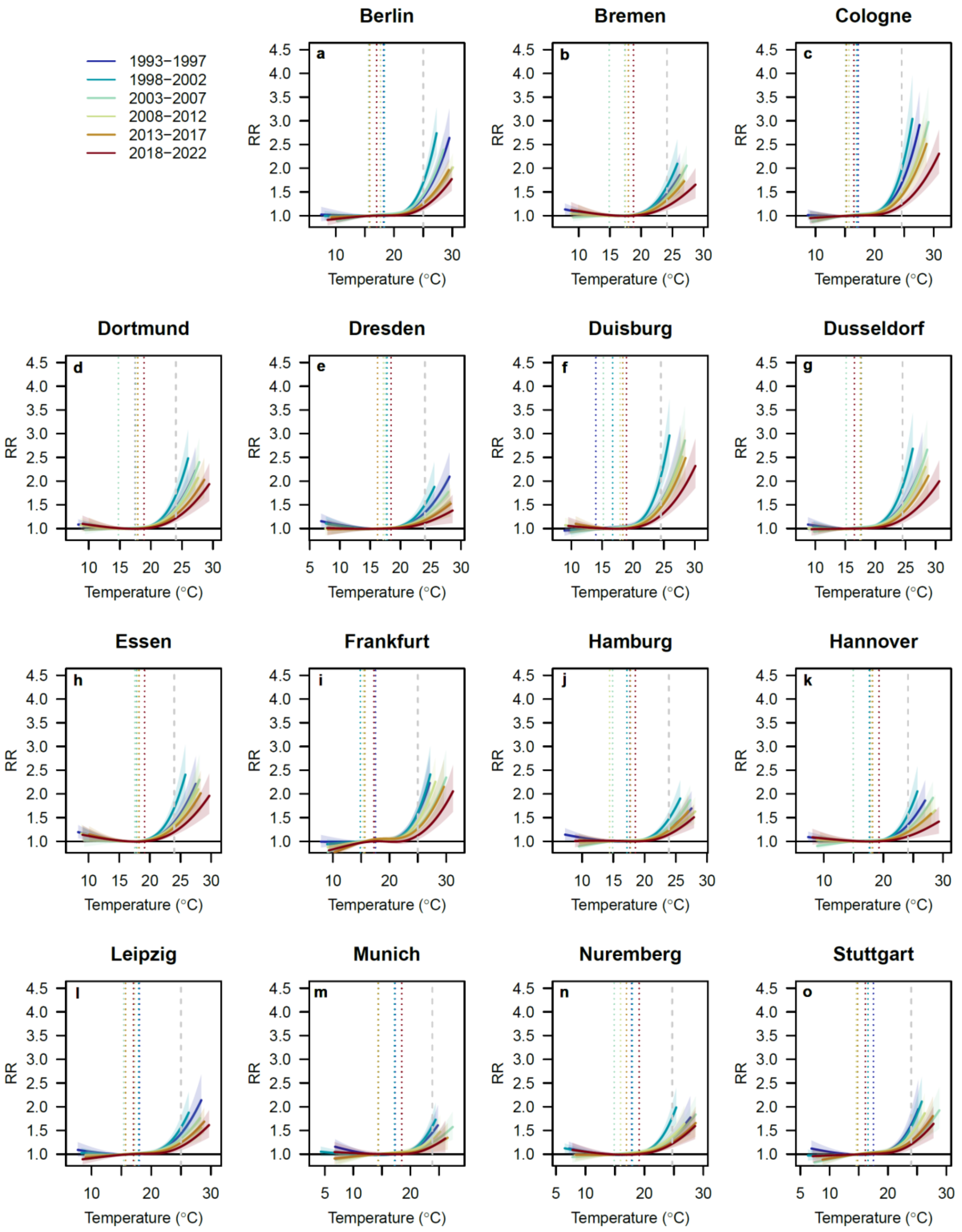

Supplementary Fig. 2 Overall cumulative temperature-mortality associations by city and subperiod, based on best linear unbiased predictors (BLUPs) from final meta-regression model (Supplementary Table 3), with 95% empirical confidence intervals; coloured vertical dotted lines show minimum mortality temperatures (MMTs); grey dashed lines show the 99th percentile temperatures from the coolest subperiod.

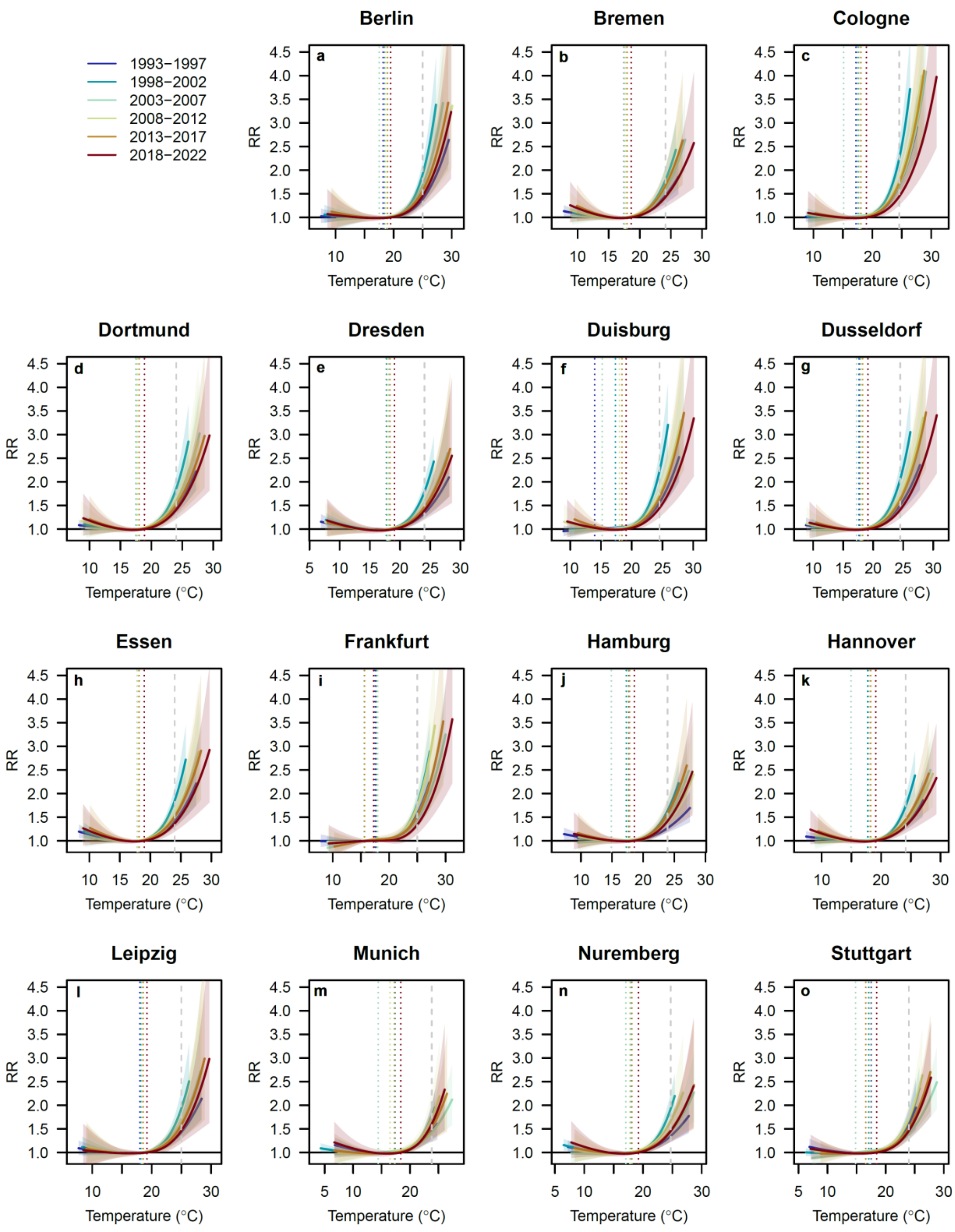

Supplementary Fig. 3 Overall cumulative temperature-mortality associations by city and subperiod, based on counterfactual best linear unbiased predictors (BLUPs), computed from final meta-regression model by fixing city-specific LE at level of first period (1993-1997); other specifications as Supplementary Fig. 2.

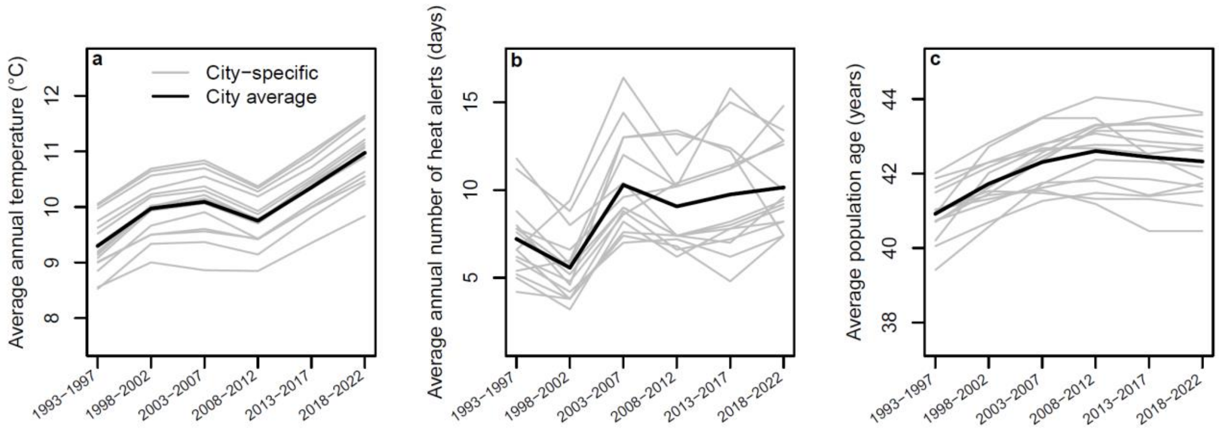

Supplementary Fig. 4 Meta-predictors included in final meta-regression model (see Supplementary Table 3). a, average annual temperatures, b, average annual number of heat alerts, c, average population age for each German city (grey) and averaged across cities (black), by subperiod.

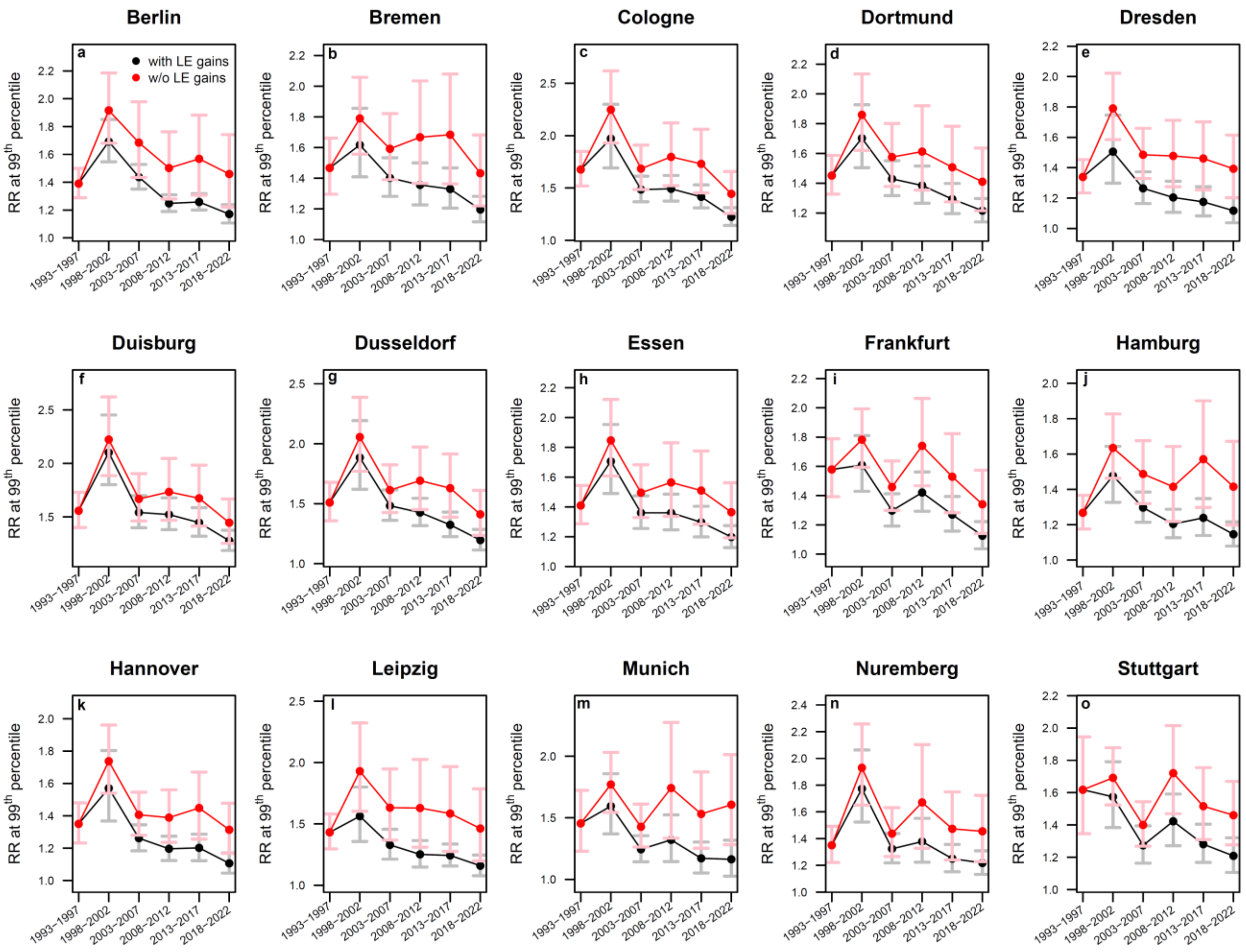

Supplementary Fig. 5 Time-varying relative risks at the 99th percentile of the daily mean temperatures in the coolest subperiod (see grey dashed lines in Supplementary Figs. 2 and 3) by city, computed based on observed life expectancy (LE) (black, factual BLUPs), and with LE fixed at 1993-1997 levels (red, counterfactual BLUPs); error bars show 95% empirical confidence intervals (n=1000 Monte Carlo samples).

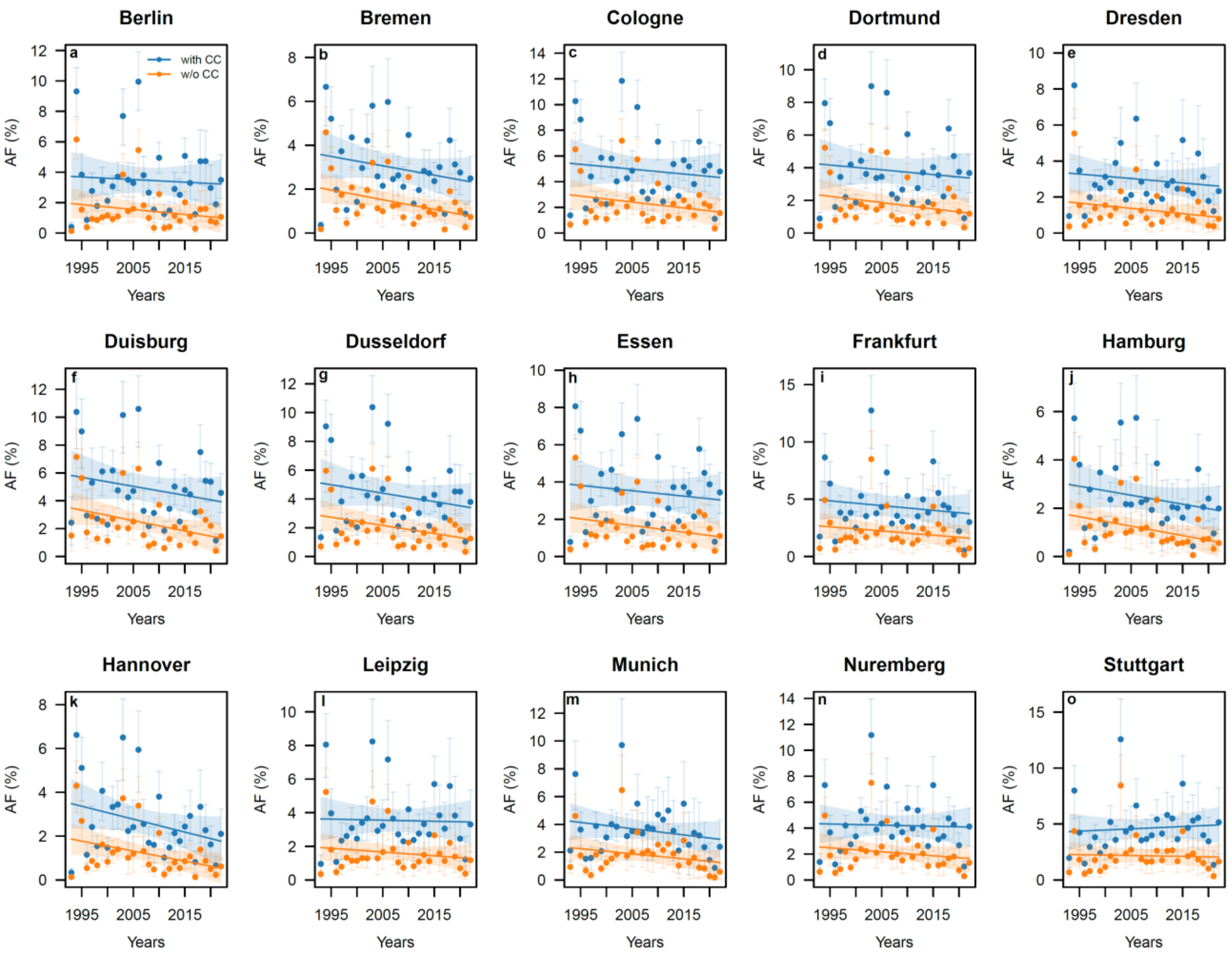

Supplementary Fig. 6 City-specific annual heat attributable fractions (AF) (as % of total warm-season mortality) for factual (with CC, blue) and best-estimate counterfactual (w/o CC, orange) temperatures, accounting for observed improvements in life expectancy (LE); lines show linear regressions (with 2*standard errors); error bars depict 95% empirical confidence intervals (n=1000 Monte Carlo samples).

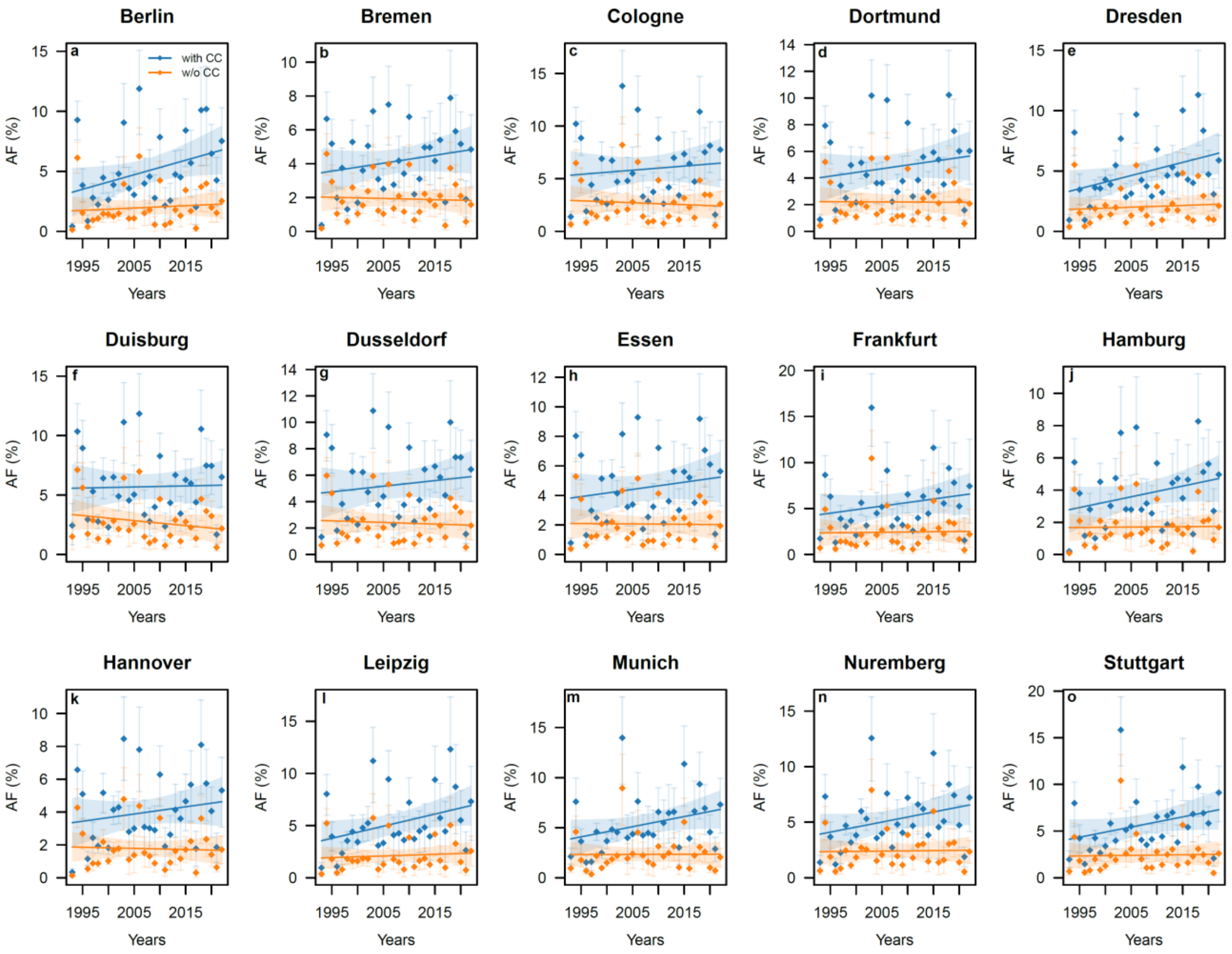

Supplementary Fig. 7 City-specific annual heat attributable fractions (AF) (as % of total warm-season mortality) for factual (with CC, blue) and best-estimate counterfactual (w/o CC, orange) temperatures, fixing city-specific life expectancy (LE) at level of first period (1993-1997). ('w/o LE improvements'); lines show linear regressions (with 2*standard errors); error bars depict 95% empirical confidence intervals (n=1000 Monte Carlo samples).

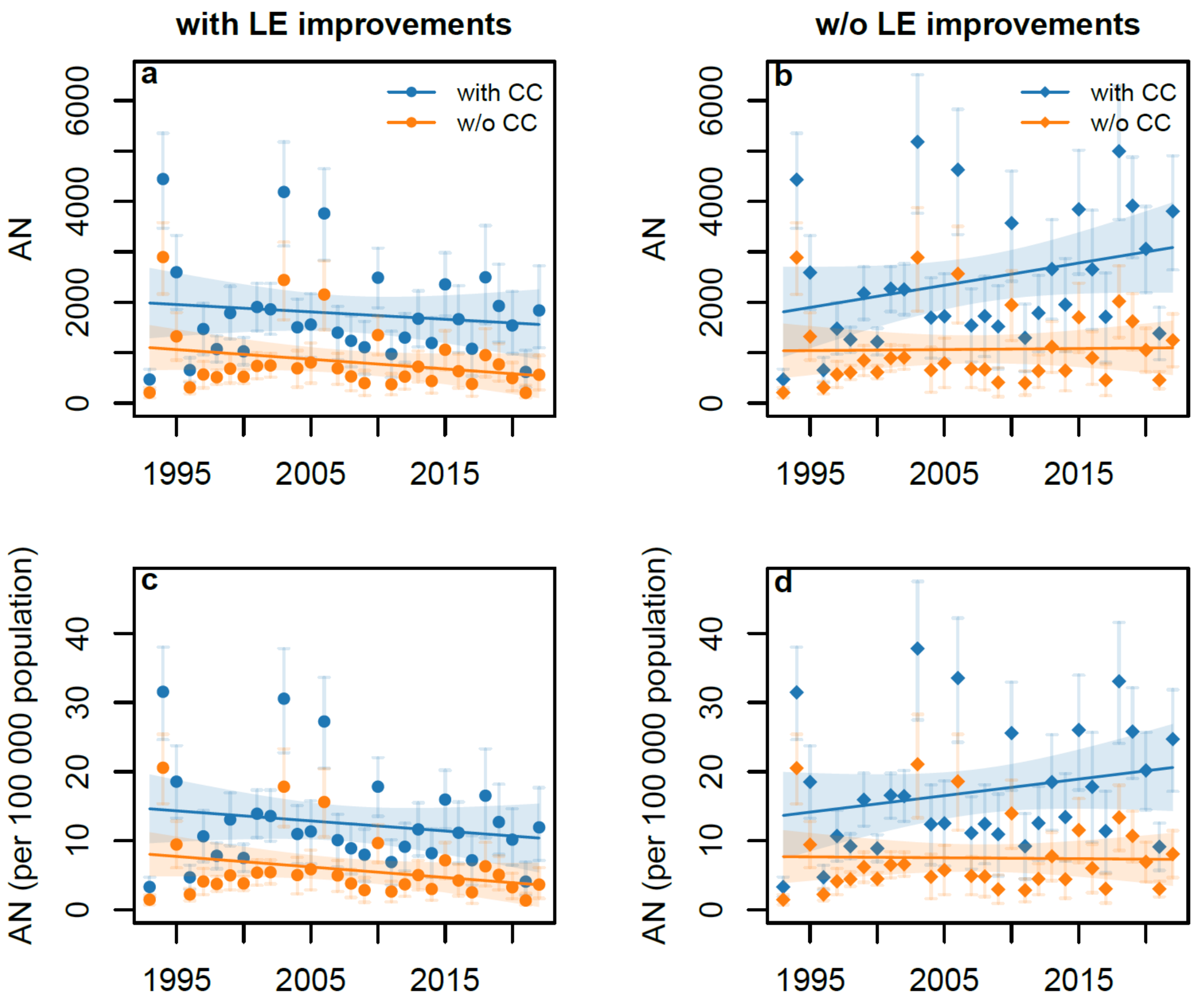

Supplementary Fig. 8 Annual heat-related excess deaths (AN) (summed across cities) (**a, b**) and mortality rates (per 100 000 population) (**c, d**) for factual (with CC, blue) and counterfactual (w/o CC, orange) temperatures, with/without considering observed improvements in life expectancy (LE); lines show linear regressions (with 2*standard errors); error bars depict 95% empirical confidence intervals (n=1000 Monte Carlo samples).

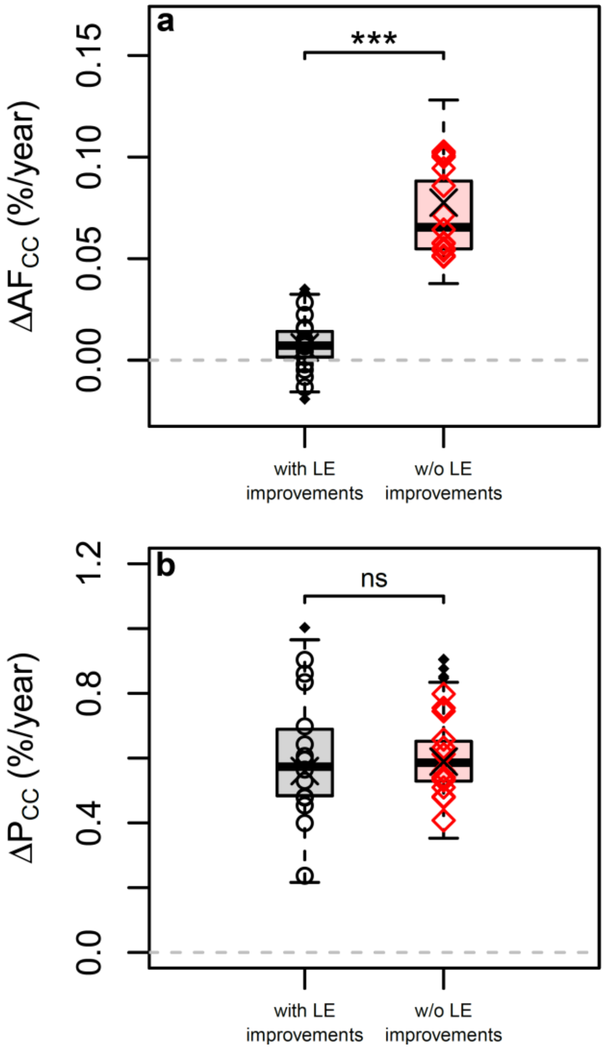

Supplementary Fig. 9 City-specific linear temporal trends of heat-related mortality attributable to climate change, with (black)/without (red) considering observed improvements in life expectancy (LE), **a**, Changes in percentage points of heat-related mortality (AF_{CC}). **b**, Changes in the relative proportion of attributable mortality (P_{CC}). Black crosses depict linear regression coefficients from pooled results in Fig. 4; boxplots show median (central line), upper and lower quartiles (box), 1.5xinterquartile range (whiskers), outliers (black dots) of all city-specific results, taking into account counterfactual temperature uncertainty (cf. Supplementary Figs. 10 and 11); statistical significance of differences in trends was assessed with a two-sided Wilcoxon rank test (***: p<0.001; ns: p=0.51; combined n=(15 cities x 9 counterfactual temperature samples) x 2=270).

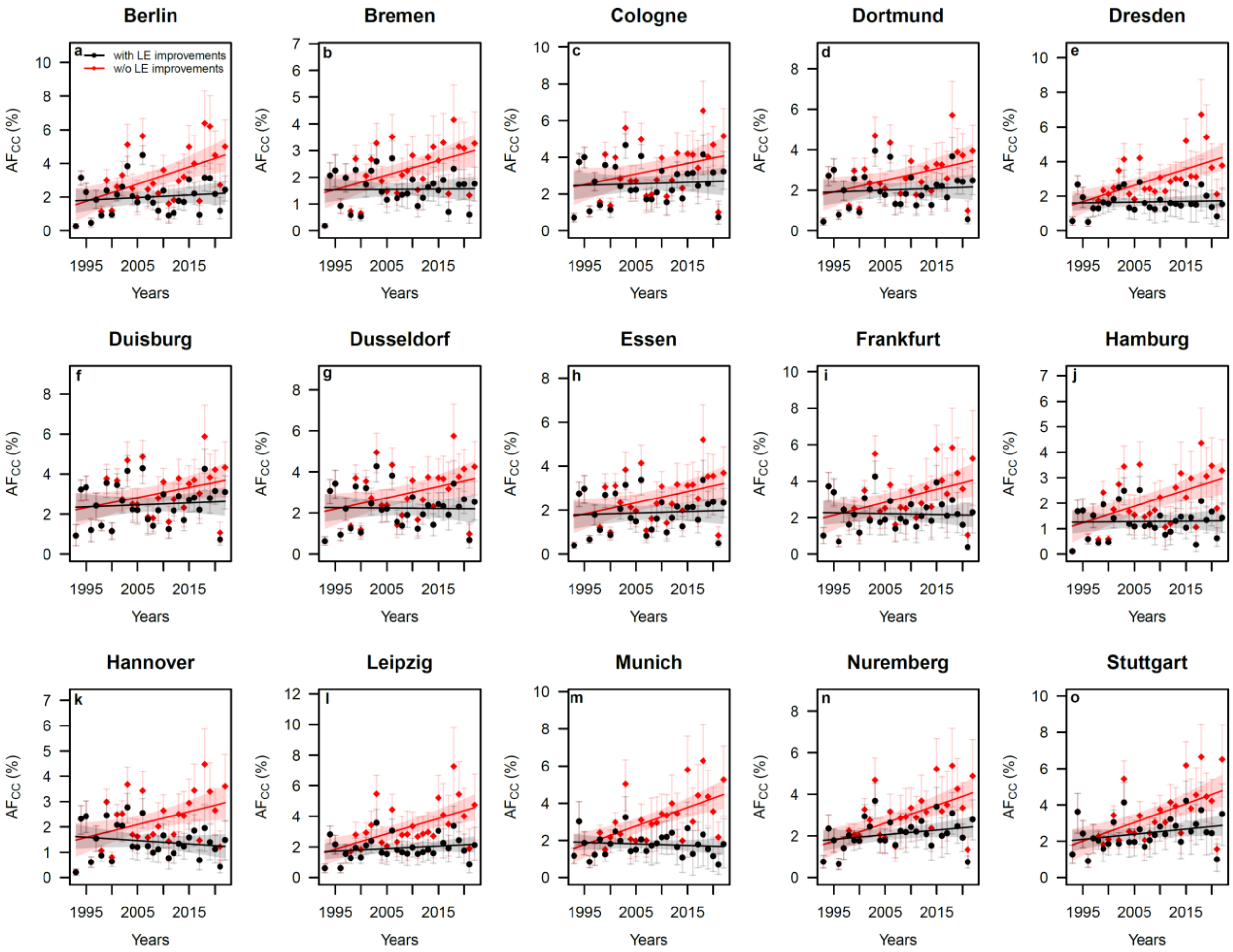

Supplementary Fig. 10 City-specific attributable fractions (AF_{CC}) (% of total warm-season mortality) computed as the difference between factual and counterfactual estimates (cf. Supplementary Figs. 6 and 7), with/without accounting for observed improvements in life expectancy (LE) lines show linear regressions (with 2*standard errors); error bars depict 95% empirical confidence intervals (n=1000 Monte Carlo samples).

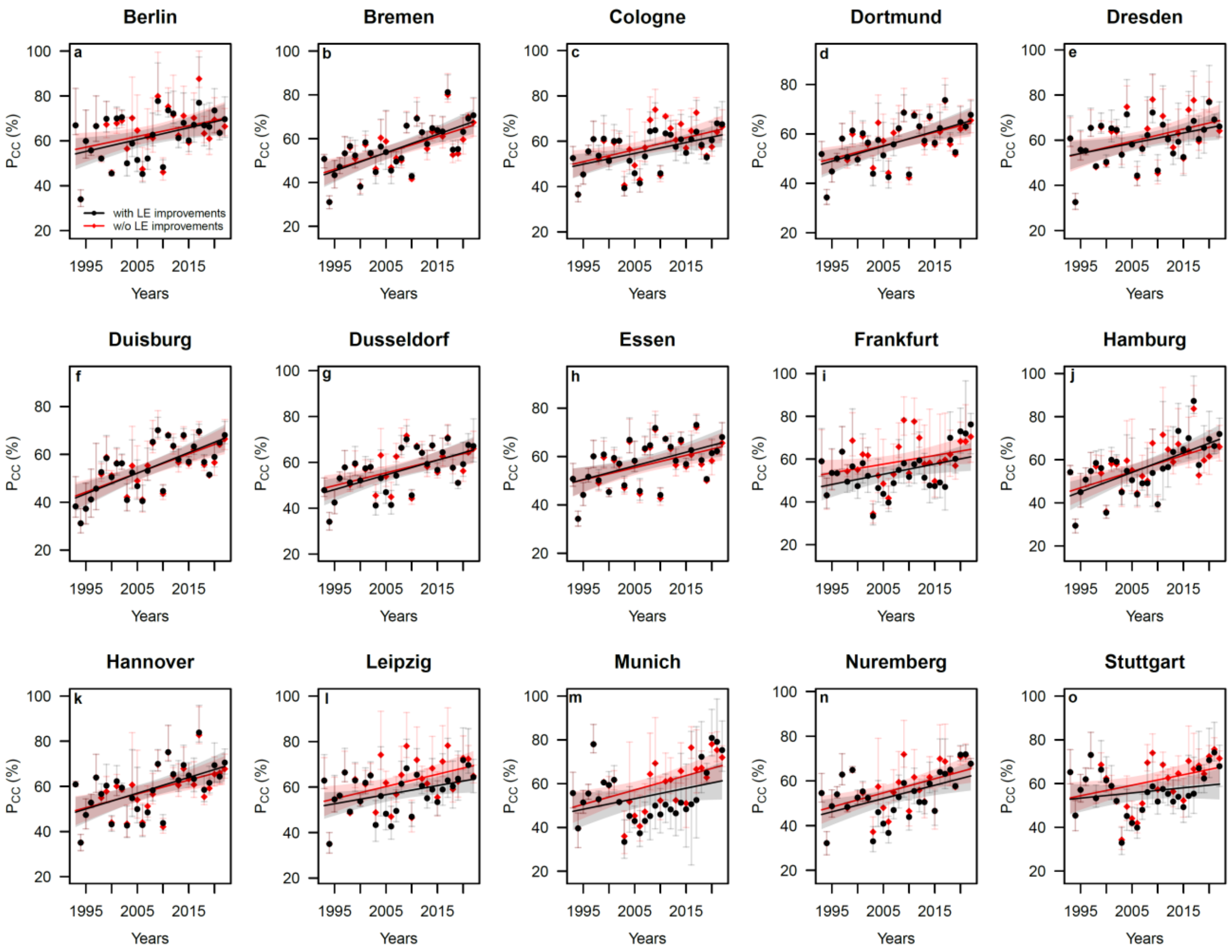

Supplementary Fig. 11 City-specific relative proportions (P_{CC}) of the observed (factual) heat-related excess mortality attributable to climate change, with/without accounting for observed improvements in life expectancy (LE); lines show linear regressions (with 2*standard errors); error bars depict 95% empirical confidence intervals (n=1000 Monte Carlo samples).

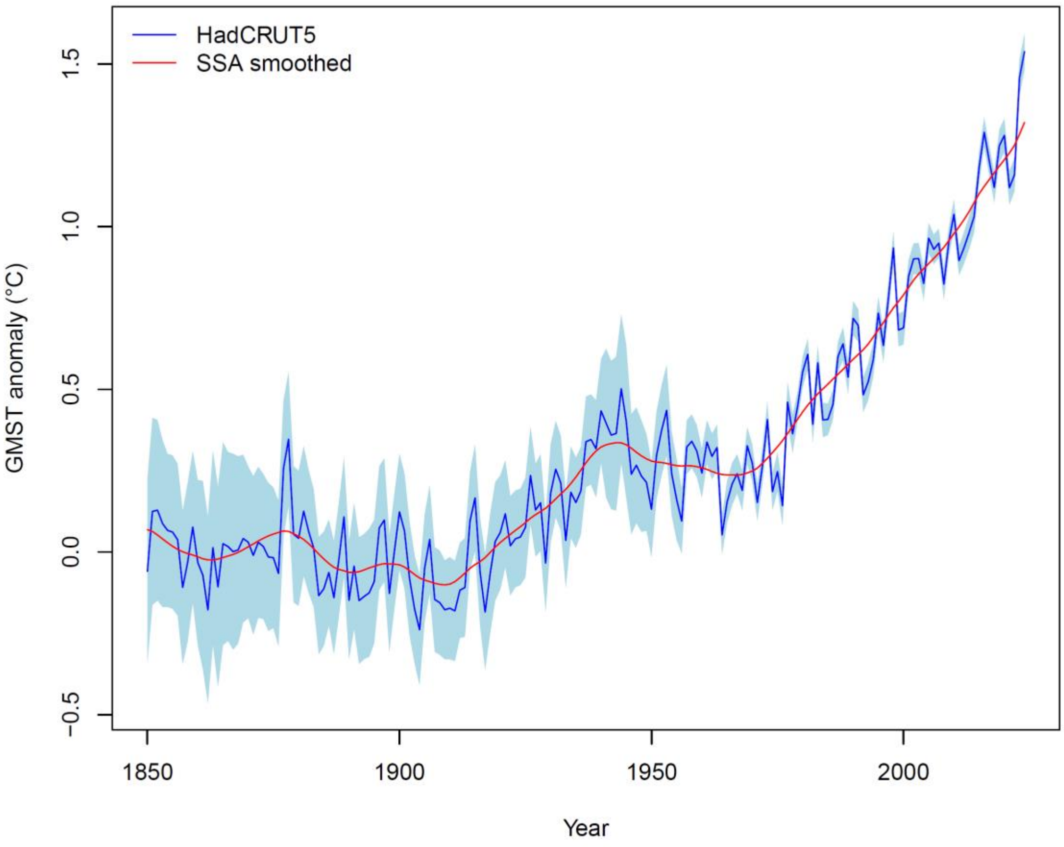

Supplementary Fig. 12 Annual global mean surface temperature (GMST) anomaly (pre-industrial reference period: 1850-1900) derived from HadCRUT5 (blue line) with 95% confidence interval (blue shading); smoothed data (red) is based on singular spectrum analysis (SSA) smoothing algorithm.

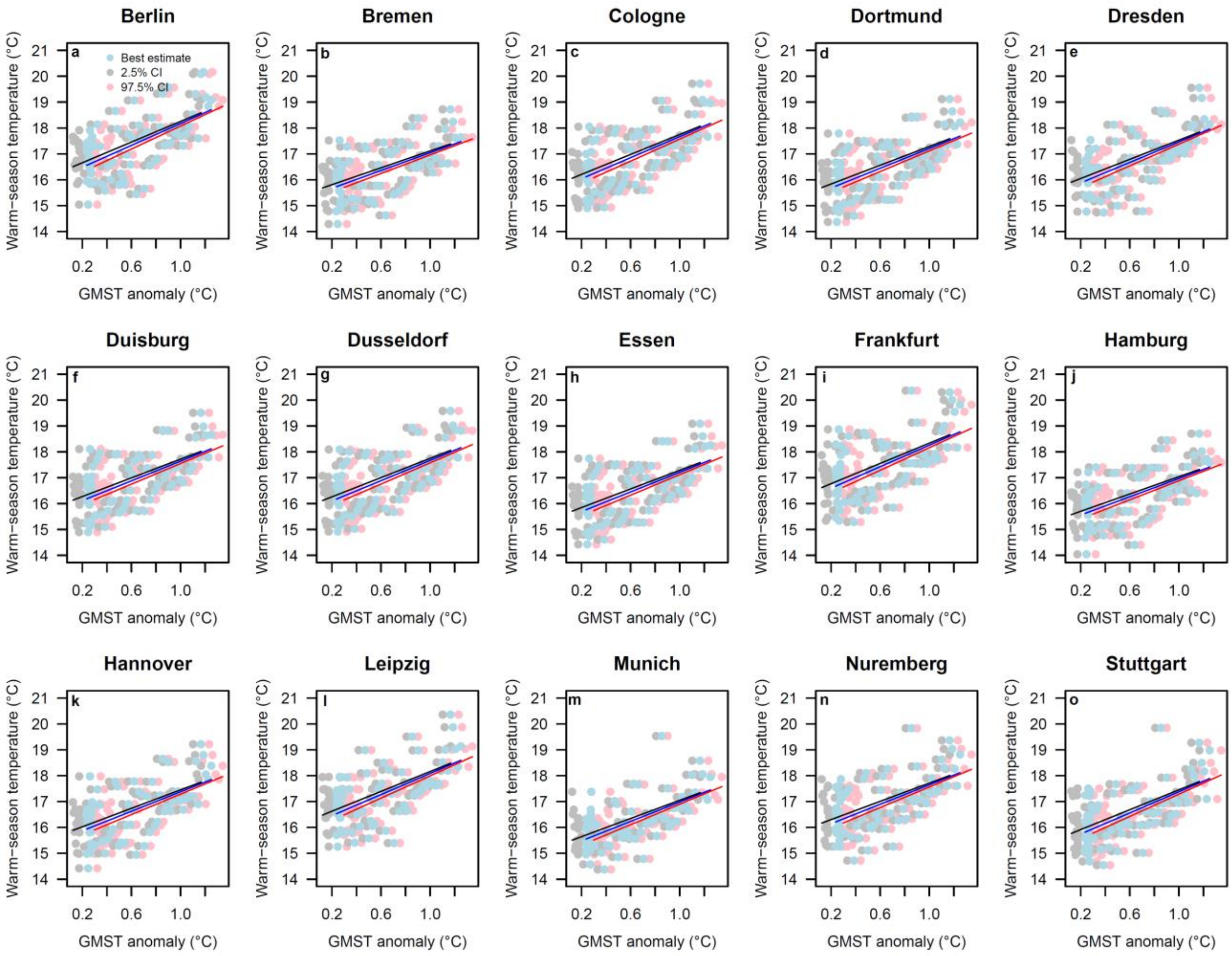

Supplementary Fig. 13 Linear regressions to derive local scaling coefficients between city-specific average warm-season (Jun-Sep) temperatures and smoothed annual global mean surface temperature (GMST) anomalies (pre-industrial reference period: 1850-1900); best estimates (blue) as well as lower (grey) and upper (red) bounds of 95% confidence intervals of HadCRUT5 GMST data are depicted.

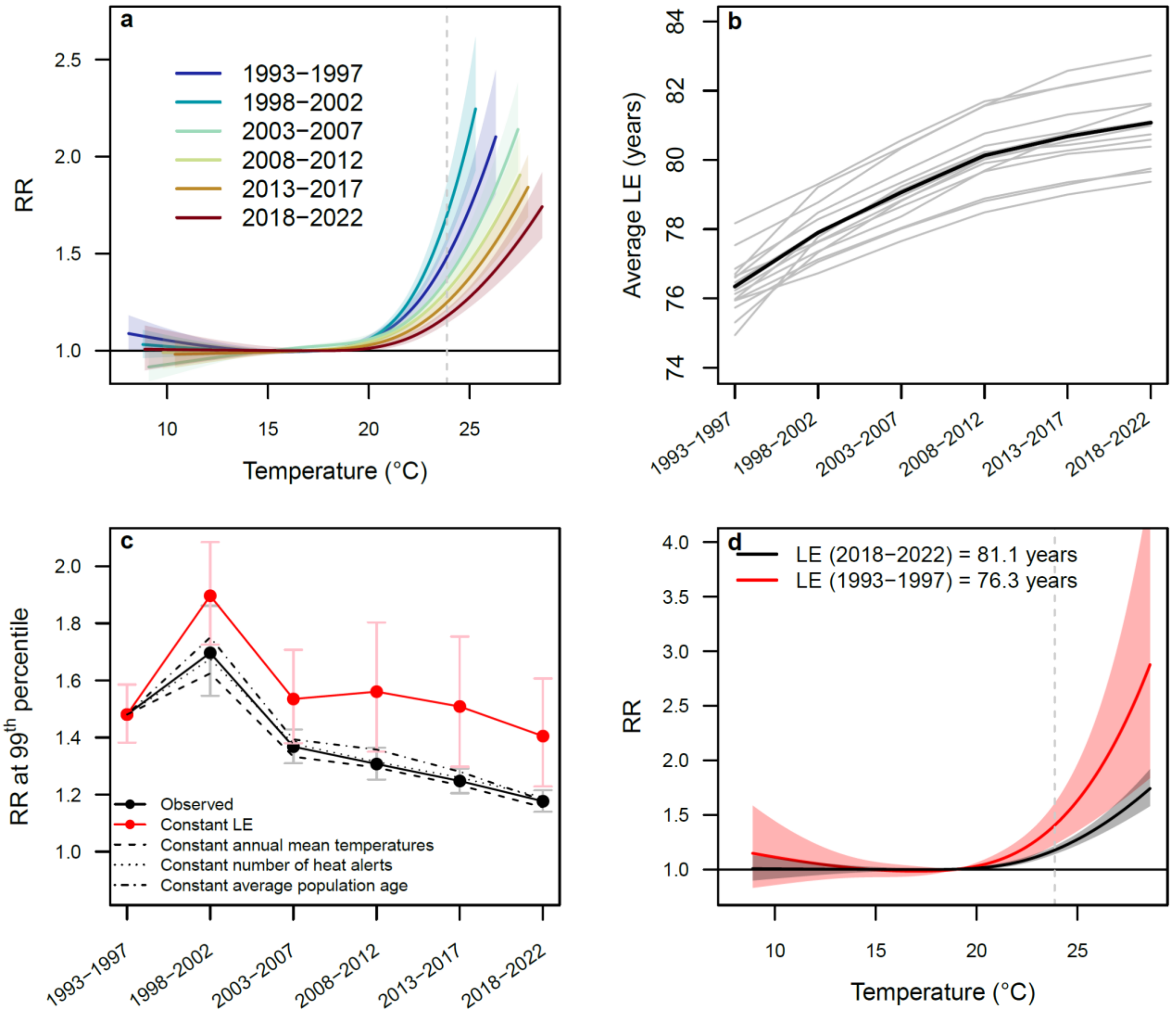

Supplementary Fig. 14 As Fig. 2, but showing results for absolute average temperatures. **a**, Pooled cumulative temperature-mortality associations by subperiod derived from final meta-regression model (Supplementary Table 3). **b**, Average LE by subperiod for each German city (grey) and averaged across cities (black). **c**, Relative risk (RR) at the 99th percentile of temperatures (vertical dashed line in a); black dots: observed RR, red dots: predicted RR assuming no change in LE (fixed at 1993-1997 average), dashed and dotted black lines: predicted RR assuming no change in the other meta-predictors. **d**, Pooled cumulative temperature-mortality association for 2018-2022, based on observed LE (black) and assuming LE had not improved since 1993-1997 (red). Error bands/bars (in a, c, d) correspond to 95% empirical confidence intervals (n=1000 Monte Carlo samples).
