# Supplementary material for: Improvements in life expectancy mask rising trends in heat-related excess mortality attributable to climate change
Source: Nat Commun. 2025 Nov 26;16:11632. doi: 10.1038/s41467-025-66681-0 (PMC12748886; doi:10.1038/s41467-025-66681-0)
Supplement: Supplementary file 2 — Reporting Summary [file 41467_2025_66681_MOESM2_ESM.pdf]

nature portfolio

Corresponding author(s): Veronika Huber

Last updated by author(s): Oct 10, 2025

Reporting Summary

Nature Portfolio wishes to improve the reproducibility of the work that we publish. This form provides structure for consistency and transparency in reporting. For further information on Nature Portfolio policies, see our Editorial Policies and the Editorial Policy Checklist.

Statistics

For all statistical analyses, confirm that the following items are present in the figure legend, table legend, main text, or Methods section.

n/a	Confirmed	
☐	☒	The exact sample size (*n*) for each experimental group/condition, given as a discrete number and unit of measurement
☒	☐	A statement on whether measurements were taken from distinct samples or whether the same sample was measured repeatedly
☐	☒	The statistical test(s) used AND whether they are one- or two-sided<br>*Only common tests should be described solely by name; describe more complex techniques in the Methods section.*
☐	☒	A description of all covariates tested
☒	☐	A description of any assumptions or corrections, such as tests of normality and adjustment for multiple comparisons
☐	☒	A full description of the statistical parameters including central tendency (e.g. means) or other basic estimates (e.g. regression coefficient) AND variation (e.g. standard deviation) or associated estimates of uncertainty (e.g. confidence intervals)
☐	☒	For null hypothesis testing, the test statistic (e.g. *F*, *t*, *r*) with confidence intervals, effect sizes, degrees of freedom and *P* value noted<br>*Give P values as exact values whenever suitable.*
☒	☐	For Bayesian analysis, information on the choice of priors and Markov chain Monte Carlo settings
☒	☐	For hierarchical and complex designs, identification of the appropriate level for tests and full reporting of outcomes
☒	☐	Estimates of effect sizes (e.g. Cohen's *d*, Pearson's *r*), indicating how they were calculated

Our web collection on statistics for biologists contains articles on many of the points above.

Software and code

Policy information about availability of computer code

Data collection	We partly used custom code to extract city-specific temperature series from the gridded ERA5 product. The code is available via GitHub (https://github.com/LAST-EBD/Consultas/tree/master/2025/Febrero/VH.)
Data analysis	All R code used for data analysis and plotting (R version 4.4.1, with packages dlnm 2.4.7 and mixmeta 1.2.0) is available via GitHub (https://github.com/veronikahuber/TrendAttribution) and deposited in zenodo (https://doi.org/10.5281/zenodo.17303702).

For manuscripts utilizing custom algorithms or software that are central to the research but not yet described in published literature, software must be made available to editors and reviewers. We strongly encourage code deposition in a community repository (e.g. GitHub). See the Nature Portfolio guidelines for submitting code & software for further information.

Data

Policy information about availability of data

All manuscripts must include a data availability statement. This statement should provide the following information, where applicable:

- Accession codes, unique identifiers, or web links for publicly available datasets
- A description of any restrictions on data availability
- For clinical datasets or third party data, please ensure that the statement adheres to our policy

The city-specific daily mean temperature data was derived from gridded ERA5-Land reanalysis 2-m air temperature data available at the Copernicus Climate Data Store (https://cds.climate.copernicus.eu/datasets/reanalysis-era5-land?tab=download). Data of HADCRUT global mean surface temperature (version 5.0.2.0) with

monthly resolution can be downloaded from https://www.metoffice.gov.uk/hadobs/hadcrut5/. Demographic and socio-economic indicators for Germany can be accessed at https://www.inkar.de/. All-cause mortality data was obtained from the Research Data Centre (RDC) of the Statistical Offices of the German Federal States, which grant restricted data access to ensure statistical confidentiality and factual anonymity. The RDC provides data access upon conclusion of a user contract to institutions of higher education or other institutions tasked with independent scientific research, usually for a duration of three years (for details see https://www.forschungsdatenzentrum.de/en/terms-use).

Research involving human participants, their data, or biological material

Policy information about studies with human participants or human data. See also policy information about sex, gender (identity/presentation), and sexual orientation and race, ethnicity and racism.

Reporting on sex and gender	The only data relevant to the concept of sex/gender is the annual percentage of women in the population, as defined in the administrative data we accessed. Yet, this indicator turned out to be a non-significant predictor of the temperature-mortality associations studied, despite the ample knowledge on the sex-specific differences in heat-related mortality risks. Our main analysis was not stratified by sex, because we did not have access to daily mortality data separately for men and women.
Reporting on race, ethnicity, or other socially relevant groupings	Our analysis is based on aggregated population data. We have not included any data referring to race or ethnicity.
Population characteristics	Our analysis is based on aggregated population data. The analysis is not stratified by age.
Recruitment	Not relevant for our study
Ethics oversight	Not required for our study, given the use of death count data without risk of re-identification of individuals.

Note that full information on the approval of the study protocol must also be provided in the manuscript.

Field-specific reporting

Please select the one below that is the best fit for your research. If you are not sure, read the appropriate sections before making your selection.

☐ Life sciences ☐ Behavioural & social sciences ☒ Ecological, evolutionary & environmental sciences

For a reference copy of the document with all sections, see nature.com/documents/nr-reporting-summary-flat.pdf

Ecological, evolutionary & environmental sciences study design

All studies must disclose on these points even when the disclosure is negative.

Study description	Our study mainly applies methods from environmental epidemiology. The epidemiological methods combine quasi-Poisson regression methods with mixed-effect meta-regression models, in a longitudinal design.
Research sample	The dataset represents approximately 1.5 Mio deaths and can be considered representative of the German urban population.
Sampling strategy	We used all mortality data accessible at the time of initating the analysis. No sample-size calculation were performed.
Data collection	Data was obtained from the Research Data Centre (RDC) of the Statistical Offices of the German Federal States, with corresponding administrative data collection procedures.
Timing and spatial scale	We use daily mortality data over the period 1993-2022, from 15 major cities (with a population > 500 000 inhabitants) . The daily temperature data used covers 1950-2022.
Data exclusions	No data was excluded.
Reproducibility	Our data analysis included several sensitivity analyses to ensure that the results did no depend on the model assumptions and parameter choices made.
Randomization	No randomization was done. The first-stage epidemiological analysis controlled for day of the week and seasonal as well as long-term trends. The second-stage meta-regression models included several demographic and socio-economic meta-predictors associated with spatio-temporal differences in temperature-mortality associations.
Blinding	Blinding is not relevant to our study, because it does not include any allocations to pre-defined groups.

Did the study involve field work? ☐ Yes ☒ No

Reporting for specific materials, systems and methods

We require information from authors about some types of materials, experimental systems and methods used in many studies. Here, indicate whether each material, system or method listed is relevant to your study. If you are not sure if a list item applies to your research, read the appropriate section before selecting a response.

Materials & experimental systems

n/a	Involved in the study
☒	☐ Antibodies
☒	☐ Eukaryotic cell lines
☒	☐ Palaeontology and archaeology
☒	☐ Animals and other organisms
☒	☐ Clinical data
☒	☐ Dual use research of concern
☒	☐ Plants

Methods

n/a	Involved in the study
☒	☐ ChIP-seq
☒	☐ Flow cytometry
☒	☐ MRI-based neuroimaging

Plants

Seed stocks	*Report on the source of all seed stocks or other plant material used. If applicable, state the seed stock centre and catalogue number. If plant specimens were collected from the field, describe the collection location, date and sampling procedures.*
Novel plant genotypes	*Describe the methods by which all novel plant genotypes were produced. This includes those generated by transgenic approaches, gene editing, chemical/radiation-based mutagenesis and hybridization. For transgenic lines, describe the transformation method, the number of independent lines analyzed and the generation upon which experiments were performed. For gene-edited lines, describe the editor used, the endogenous sequence targeted for editing, the targeting guide RNA sequence (if applicable) and how the editor was applied.*
Authentication	*Describe any authentication procedures for each seed stock used or novel genotype generated. Describe any experiments used to assess the effect of a mutation and, where applicable, how potential secondary effects (e.g. second site T-DNA insertions, mosiacism, off-target gene editing) were examined.*
